# Use of ‘eradication’ in HIV cure-related research: a public health debate

**DOI:** 10.1186/s12889-018-5141-2

**Published:** 2018-02-13

**Authors:** Karine Dubé, Stuart Luter, Breanne Lesnar, Luke Newton, Jerome Galea, Brandon Brown, Sara Gianella

**Affiliations:** 1 UNC Gillings School of Global Public Health, 4108 McGavran-Greenberg Hall, Chapel Hill, NC 27516 USA; 2 Department of Global Health and Social Medicine, Harvard Medical School, Boston, MA 02115 USA; 3 Center for Healthy Communities, Department of Social Medicine and Population Health, University of California Riverside School of Medicine, 3333 14th Street, Riverside, CA 92501 USA; 4 University of San Diego School of Medicine, 9500 Gilman Drive #0679, La Jolla, CA 92093 USA

**Keywords:** HIV cure, Eradication, Public health, Terminology

## Abstract

**Background:**

The landscape of Human Immunodeficiency Virus (HIV) research has changed drastically over the past three decades. With the remarkable success of antiretroviral treatment (ART) in decreasing AIDS-related mortality, some researchers have shifted their HIV research focus from treatment to cure research. The HIV cure research community often uses the term *eradication* to describe the science, and talks about eradicating the virus from the body. In public discourse, the term eradication could be conflated with disease eradication at the population level. In this paper, we call for a reframing of HIV cure research as control, as it is a more accurate descriptor and achievable goal in the foreseeable future.

**Discussion:**

The properties of HIV are discordant with eradicability standards at both the individual level (as a clinical concept), and at the population level (as a public health concept). At the individual level, true eradication would necessitate absolute elimination of all latent HIV reservoirs from the body. Current HIV cure-related research strategies have proven unsuccessful at accurately quantifying, let alone eliminating these reservoirs. At the population level, eradication implies the permanent global reduction of HIV to zero new cases and to zero risk for future cases. Given the absence of an efficacious HIV vaccine and the impracticality and unethicality of eliminating animal reservoirs, global eradication of HIV is highly implausible. From a public health perspective, HIV eradication remains an elusive goal.

**Conclusion:**

The term ‘eradication’ is a misleading description of current HIV cure-related research. Instead, we call for the use of more realistic expressions such as ‘sustained virologic HIV suppression (or control)’ or ‘management of HIV persistence’ to describe HIV cure-related research. Using these terms reorients what HIV cure science can potentially achieve in the near future and avoids creating unrealistic expectations, particularly among the millions of people globally who live with HIV.

## Background

An estimated 36.7 (30.8–42.9) million people live with HIV worldwide, and approximately 1.8 (1.6–2.1) million new infections occurred in 2016 [1]. Global scale-up of HIV antiretroviral treatment (ART) increased the life expectancy of people living with HIV, contributing to a 48% reduction in AIDS-related mortality between 2005 and 2016 [1, 2]. However, ART does not remove replicative HIV from the body and is not a cure [3]. Barriers to an HIV cure include the persistence of quiescent HIV reservoirs that are not sensitive to or reached by ART nor visible to the immune system. The last decade has seen an increase in biomedical HIV cure research [4]. The U.S. Food and Drug Administration (FDA) defines HIV cure research as: “any investigation that evaluates: 1) a therapeutic intervention or approach that controls or eliminates HIV infection to the point that no further medical interventions are needed to maintain health; and 2) preliminary scientific concepts that might ultimately lead to such a therapeutic intervention” [5]. HIV cure research involves a variety of biomedical approaches (e.g. latency-reversing compounds; early and intensified HIV therapy; immune-based strategies and therapeutic vaccines; gene editing; stem cell transplants; or combined approaches) [4]. Two main pathways to achieving sustained ART-free HIV suppression are under investigation: 1) removal of all replication-competent HIV reservoirs from the body (classic biomedical cure), and 2) control of viral rebound in the absence of ART without complete elimination of HIV (‘functional cure’) [6].

Eradication is a distinct concept at the individual level and at the population level. On an individual patient level, HIV cure would mean eradication of the virus from the body. HIV cure remains an aspirational goal for most people living with HIV. One person, Timothy Ray Brown, has been cured of HIV infection [7], but the science of HIV cure research has not progressed to the point where eradication of HIV reservoirs from the body (a cure) is possible. The primary intent of most HIV cure interventions remains long-term virologic suppression of HIV in the absence of ART. Most HIV cure research approaches under clinical investigation only have the capacity to reduce but not eliminate the reservoir size or augment the immune system’s ability to control HIV while off ART. On a population level and as a public health concept, eradication is defined as the permanent reduction to zero of the worldwide *incidence* of a disease [8]. For example, this would mean the elimination of transmission and disease, as seen with smallpox eradication. From a public health perspective, eradication is inherently an incidence concept, not a cure concept. Moreover, HIV does not meet the criteria for disease eradicability set forth by the World Health Organization (WHO) and the International Task Force for Disease Eradication (ITFDE) [8, 9].

While the HIV cure research community uses the term *eradication* as a clinical concept, it could easily be misinterpreted as disease eradication at a population level in public discourse. Scientific groups such as the Collaborative HIV Eradication of Viral Reservoirs (CHERUB), the amfAR Research Consortium on HIV Eradication (ARCHE) and the Collaboratory of AIDS Researchers for Eradication (CARE) are dedicated to finding a clinical cure for HIV infection. The biomedical HIV cure research literature is also rich with examples of potential ‘eradication therapies’ for HIV infection [10–15]. The term *eradication* is used to galvanize interest and funding in the science. Elimination would be a better term to describe the complete disappearance of HIV from the body as an aspirational cure goal. Instead of eradication, we also suggest the use of expressions such as ‘long-term virologic suppression (or control) off therapy’, or ‘management of HIV persistence’ [6, 16]. Control of HIV makes much more sense as it is a well-documented concept and can be linked appropriately to viral replication [17, 18]. From a public health perspective, when the term eradication is used loosely, it can easily be conflated with HIV elimination or control on a global scale. The public should be informed that HIV cure research will not lead to disease eradication on a population level. Using the term ‘control’ on a clinical and population levels provides a reorientation of what HIV cure science can potentially achieve in the foreseeable future and avoids creating false hopes and expectations [19], particularly among the millions of people globally who live with HIV.

## Discussion

The term eradication borrows its origins from the Latin word *eradicat*, meaning “torn up by the roots” [20]. In HIV cure-related research, the term eradication is used to denote the complete elimination of all replication-competent forms of the virus (*eradicating* or *sterilizing* cure) [10]. Relatedly, language used to describe the science provides an opportunity to educate the community and potential volunteers regarding what can be accomplished to avoid the overestimation of possible medical benefits [19]. Careful examination of HIV cure-related research terminology reveals the terms used are complex, and oftentimes considered only in narrow biomedical contexts in isolation from the social contexts of disease [17, 18]. HIV cure-related research carries hopes and misconceptions. Using language such as cure or eradication risks creating public misapprehension about incremental research developments [18]. This paper builds on the literature interrogating the use of language in HIV cure-related research [17, 18, 21, 22] and on a rich history of questioning language used in the scientific literature in relation to HIV (e.g. for example, gay-related immune deficiency (GRID) was renamed acquired immune deficiency virus (AIDS) in 1982).

### At the individual (clinical) level, the expression *HIV control* is more descriptive than *HIV eradication*

Anthony Fauci, Director of the National Institute of Allergies and Infectious Diseases (NIAID), stated: “the complete eradication or elimination of HIV from an HIV-infected individual would be extremely difficult to achieve. At worst, it would be impossible. At best, it would be achievable in a very small subset of the HIV-infected population” [6].

At the individual level, HIV control may be considered a more descriptive endpoint than eradication. Complete removal of all HIV remnants is an extremely high bar to achieve [18]. The fundamental biology of integrated proviral HIV deoxyribonucleic acid (DNA) makes it nearly impossible to know if all inducible virus has been removed [13]. Another obstacle to curing HIV is ongoing viral replication, even in the presence of ART [13]. The suspected mechanism of this residual ongoing replication is direct cell-to-cell transmission, in which HIV passes from an infected cell to an adjoining uninfected cell [13]. Low-level viral replication escapes the effect of ART and contributes to the long-term persistence of HIV [13].

Timothy Ray Brown, the Berlin patient, is the only person currently cured of HIV [7]. His cure involved a set of circumstances that have yet to be replicated. Following a relapsed acute myeloid leukemia (AML), he received two stem cell transplants from a donor homozygous for the CCR5 delta32 deletion. His initial case was described as ‘long-term control of HIV’ [23]. Mr. Brown remained without replication-competent provirus for over 10 years. However, a recent study found trace amounts of replication-incompetent HIV nucleic acid in his body (researchers did not exclude the possibility of false positive results) [24]. Even with the complete elimination of replication-competent HIV, finite particles of HIV may persist in the body, excluding true viral eradication.

Other case histories are similarly illustrative of the complexity of the issue and the need for precise language. Even small HIV reservoirs do not guarantee infection control when off ART [25]. A recent case study showed an adult treated during early (Fiebig stage I) HIV infection with prolonged HIV suppression, followed by viral rebound. In this study, HIV treatment in the earliest stage of HIV infection resulted in a small number of latently infected cells but did not eliminate replicative HIV reservoirs [26]. The Mississippi child represents another unusual case of prolonged HIV suppression with small HIV reservoirs [27]. The perinatally infected infant tested positive at birth for HIV and immediately received ART. Her therapy was interrupted against medical advice when she was between 18 and 23 months old. The Mississippi child remained with undetectable viremia for approximately 27 months, until HIV was detected in her blood at 46 months of age [27]. Because she started ART very early, it is possible that her HIV reservoir size was negligible; however, the return of HIV after a prolonged period of quiescent virus is consistent with the HIV latency hypothesis [13]. Moreover, two patients from Boston underwent allogeneic bone marrow transplants to treat lymphoma, resulting in a reduction of their replication-competent HIV reservoir size [28]. After a prolonged period of ART-free suppression, their virus also rebounded [29].

The Mississippi child and the Boston patients are emblematic cases demonstating the limits of HIV reservoir size estimations. Current assays to measure residual replication-competent HIV are not sufficiently sensitive to rule-out incomplete clearance of HIV from blood or tissues [10, 13, 30]. While some HIV cure research modalities aim to reduce the size of the inducible HIV reservoir (e.g., latency-reversing agents or stem cell transplants), prolonged ART-free suppression would require a drastic decrease in the pool of latently infected cells. These cells are extremely rare, with an average frequency of about 1 cell per 10^6^ resting CD4^+^ T cell lymphocytes [31]. The current gold standard for measuring these replication-competent HIV reservoirs is the Quantitative Viral Outgrowth Assay (QVOA). Though sensitive, the QVOA underestimates the size of the latent reservoir because some of the proviruses are not induced even after several rounds of viral outgrowth activation [32]. In the Mississippi child and the Boston patients, for example, virus inducibility from latent reservoirs could not be detected during aviremic intervals, yet subsequent viral rebounds showed the presence of reproducible virus below the limit of detection [10, 29, 33]. The QVOA could not measure all latent virus in tissue sanctuaries [34] or other reservoirs (e.g., those in the brain) [35]. Biomedical HIV cure researchers posit that only one replication-competent HIV DNA strand is needed for a total eruption of viral replication. Thus, the terms “reservoir reduction” or “viral control” may be better aligned with strategies aimed at depleting the HIV reservoir while strengthening the immune system’s ability to fight reawakening virus [35].

Much like cancer, proviral HIV is a residual disease, and a cure is limited by the persistence of rare cells [36]. Replication-competent reservoirs can exist in blood or tissue even when they cannot be measured. The axiom ‘absence of evidence is not evidence of absence’ is apt in this case. Due to the limited ability to measure latent HIV, any patient who controls HIV off treatment would still require indefinite clinical follow-up to monitor HIV reactivation [30, 37].

Moreover, most current HIV cure research strategies do not lead to a substantial reduction in the size of the HIV reservoir. For example, early ART initiation restricts, but does not eliminate HIV reservoirs [38, 39]. Latency-reversing agents have not yet shown to cause a notable reduction in the size of the replication-competent HIV reservoirs in clinical studies [40, 41]. Immunotherapeutic approaches are being investigated to facilitate viral clearance [42–44], but do not inherently result in HIV reservoir eradication, but rather the more rapid removal of reactivating cells. Scientists are also studying gene modification strategies, particularly those blocking HIV cell entry [45]. Genetically-edited cells can be reinfused into patients but to date engrafted cells represent a minority of the cell population, and viral relapse is common following ART interruption [45]. In turn, stem cell transplants involve risky procedures, such as ablative conditioning, and are only appropriate for a very small portion of people living with HIV who have an underlying disease indicating such an approach [6]. To date, no HIV cure research strategy has been able to overcome the challenge of eliminating proviral HIV latency or conferring viral suppression off ART at an individual level.

### HIV does not meet criteria for eradicability at the population level

On a global scale, eradication is often conflated with disease control, which refers to the reduction of disease burden to an acceptable level, without the expectation that the disease would disappear forever [46].

The term *eradication* in relationship to disease gained popularity at the beginning of the twentieth century, following the establishment of germ theory and the growth of public health policy focused on designing countermeasures (e.g, vaccines and antibiotics). In 1909, the Rockefeller Foundation sponsored the Sanitary Commission for the Eradication of Hookworm Disease, followed by a similar Yellow Fever Commission in 1915 [46]. After the Second World War, eradication was endorsed by the newly created WHO, with eradication efforts against malaria, smallpox, yaws, poliomyelitis and guinea worm disease (dracunculiasis) [46]. Smallpox was the first, and thus far only, disease to meet the true absolutist goal of eradication (1980), following an intense and deliberate worldwide effort to bring its incidence to zero [46]. Smallpox eradication required an efficacious vaccine conferring lifelong immunity, no animal hosts or intermediate vectors, and high symptomaticity so the disease could be identified efficiently during eradication efforts. Smallpox also had a high profile in public health in the twentieth century, and there was tremendous political will to bring its global incidence down to zero [46].

As demonstrated with smallpox, the meaning of the word *eradication* in global public health is the permanent reduction to zero new cases, and zero risk of cases [47] – or *incidence* of infection caused by a pathogen [8, 47]. Control in this context means the reduction of disease incidence, prevalence, and/or morbidity or mortality to an acceptable level, with continuous measures to keep the infection under control [8]. Elimination of infection means the reduction to zero of the incidence of disease with continuous efforts to prevent re-establishment of transmission [8]. Disease extinction signifies that the pathogen no longer exists in nature or in the laboratory [8]. By this definition, no infection meets the extinction criteria since stocks of smallpox virus remain in two laboratories in Atlanta, GA, USA, and Moscow, Russia [8].

The International Task Force for Disease Eradication (ITFDE) has established their own criteria for disease eradication. Examining over 90 diseases, the ITFDE determined that only six – dracunculiasis, poliomyelitis, mumps, rubella, lymphatic filariasis, and cysticercosis – were candidates for eradication with currently available biomedical interventions [9]. WHO subsequently called for the elimination of leprosy, onchocerciasis, and Chagas diseases as significant public health problems [8]. The criteria met by these diseases include: (1) easily diagnosed; (2) effective interventions (e.g., vaccines) exist to break the cycle of transmission; and (3) infection occurs only in humans (i.e., no animal reservoirs). Selecting a disease candidate for eradication is not only a question of scientific feasibility, but also a matter of sincere political commitment and funding [8, 9, 46]. HIV eradication on a global scale is extremely difficult, if not impossible, to achieve in the absence of a highly efficacious vaccine [48] and the permanent existence of simian reservoirs harboring immunodeficiency virus. It would be impractical to eliminate animal reservoirs, short of exterminating all non-human primates in the wild carrying potentially infectious virus. This would not only be environmentally unacceptable and unethical, but also at odds with principles of ‘one health’ and disease ecology [46]. Therefore, doubts remain as to the eradicability potential of HIV/AIDS in humans on a global scale.

### A public health perspective to HIV control

Eradication as an absolute goal may rest on an over-reliance on biomedical technologies that fail to account for the complexity of transmission [46]. To reduce the worldwide burden of HIV, it is critical to adopt public health strategies that leverage both biomedical and social interventions [49, 50]. A purely biomedical, therapeutic, or curative approach to HIV control is reductionist, failing to account for the complexity of disease. In fact, an effective response to HIV should be informed by a prediction of where the next 1000 new infections are likely to emerge, taking into account the deeper, and more intractable social, economic, behavioral, structural, and political determinants of HIV [51]. Disease control will rest on keeping up the “momentum for effective, complex, combination HIV prevention and treatment efforts over the long term” [51]. Yet scale-up of voluntary medical male circumcision remains a challenge in several African countries [52]. Additionally, we are far from reaching the 90–90-90 HIV treatment targets globally that would faciliate achieving epidemic control through reduced viral loads [53]. An over-emphasis on therapeutic and curative biomedical technologies may myopically create solutions that are inadequate and misguided to control such a profoundly social disease [49]. A cure for HIV would be a tremendous scientific achievement, but would unlikely lead to HIV elimination as the disease is entangled with many complex structural determinants. Over-excitement in the HIV cure endeavor may eclipse the myriad socio-structural factors that create vulnerability to HIV in the first place [22].

On a global level, it may be worth asking if HIV ‘eradication’ is a worthwhile endeavor, knowing that animal reservoirs exist and that zoonotic leaps may lead to recurrent outbreaks in humans much like those seen with Ebola [54]. While eradication has been a useful end goal for smallpox, with HIV it may be an example of over-confidence in biomedical approaches. From a public health perspective, it may be time to scale back the HIV eradication endeavor to focus instead on HIV control.

## Conclusion

In sum, we argued for replacing the word eradication in HIV cure science as it exists. On an individual level, complete elimination of replication-competent HIV is an extremely high bar to achieve. While the term eradication is meant as a clinical concept by the HIV cure research community, it could be conflated with disease eradication at the population level in public discourse. On a population level, HIV eradication is an elusive goal, since HIV does not meet the criteria for disease eradicability [8, 9]. Framing HIV cure research as control research may help us calibrate expectations of what is possible, at least in the short term [19]. Eradication requires perfection, setting the bar high and engendering chronic disappointment. Additionally, focusing on HIV eradication could lead to premature abandonment of a worthwhile enterprise. Further empirical research is needed to determine how people living with HIV, and other stakeholders, perceive the word eradication when discussing HIV cure research. We advocate to use expressions such as ‘sustained virologic HIV suppression (or control) off treatment’ [6] or ‘management of HIV persistence’ [16] to describe HIV cure-related research. These are more realistic and relevant endpoints. The control paradigms at individual and population levels allow us to more aptly appreciate incremental achievements in scientific research [19].
